# Bathing Adaptations in the Homes of Older Adults (BATH-OUT-2): study protocol for a randomised controlled trial, economic evaluation and process evaluation

**DOI:** 10.1186/s13063-023-07677-3

**Published:** 2024-01-22

**Authors:** Phillip J. Whitehead, Stuart Belshaw, Samantha Brady, Elizabeth Coleman, Alexandra Dean, Laura Doherty, Caroline Fairhurst, Sandra Francis-Farrell, Miriam Golding-Day, Joanne Gray, Maisie Martland, Jennifer McAnuff, Andrew McCarthy, Peter McMeekin, Natasha Mitchell, Melanie Narayanasamy, Craig Newman, Adwoa Parker, Tim Rapley, Sara Rodgers, Leigh Rooney, Rachel Russell, Laura Sheard, David Torgerson

**Affiliations:** 1 Population Health Sciences Institute, Newcastle University, Newcastle Upon Tyne, UK; 2 Centre for Rehabilitation and Ageing Research, University of Nottingham, Nottingham, UK; 3 Department of Health Sciences, University of York, York, UK; 4 Nottingham City Council, Nottingham, UK; 5 Department of Nursing, Midwifery and Health, Northumbria University Newcastle, Newcastle Upon Tyne, UK; 6 Department of Social Work, Education and Community Wellbeing, Northumbria University Newcastle, Newcastle Upon Tyne, UK; 7 Foundations, Glossop, UK

**Keywords:** Randomised controlled trial, Bathing adaptations, Older adults, Local authorities, Occupational therapy, Social care research

## Abstract

**Background:**

The onset of disability in bathing is particularly important for older adults as it can be rapidly followed by disability in other daily activities; this may represent a judicious time point for intervention in order to improve health, well-being and associated quality of life. An important environmental and preventative intervention is housing adaptation, but there are often lengthy waiting times for statutory provision. In this randomised controlled trial (RCT), we aim to evaluate the effectiveness and cost-effectiveness of bathing adaptations compared to no adaptations and to explore the factors associated with routine and expedited implementation of bathing adaptations.

**Methods:**

BATH-OUT-2 is a multicentre, two-arm, parallel-group RCT. Adults aged 60 and over who are referred to their local authority for an accessible level access shower will be randomised, using pairwise randomisation, 1:1, to receive either an expedited provision of an accessible shower via the local authority or a usual care control waiting list. Participants will be followed up for a maximum of 12 months and will receive up to four follow-ups in this duration. The primary outcome will be the participant’s physical well-being, assessed by the Physical Component Summary score of the Short Form-36 (SF-36), 4 weeks after the intervention group receives the accessible shower. The secondary outcomes include the Mental Component Summary score of the SF-36, self-reported falls, health and social care resource use, health-related quality of life (EQ-5D-5L), social care-related quality of life (Adult Social Care Outcomes Toolkit (ASCOT)), fear of falling (Short Falls Efficacy Scale), independence in bathing (Barthel Index bathing question), independence in daily activities (Barthel Index) and perceived difficulty in bathing (0–100 scale). A mixed-methods process evaluation will comprise interviews with stakeholders and a survey of local authorities with social care responsibilities in England.

**Discussion:**

The BATH-OUT-2 trial is designed so that the findings will inform future decisions regarding the provision of bathing adaptations for older adults. This trial has the potential to highlight, and then reduce, health inequalities associated with waiting times for bathing adaptations and to influence policies for older adults.

**Trial registration:**

ISRCTN Registry ISRCTN48563324. Prospectively registered on 09/04/2021.

## Administrative information

Note: The numbers in curly brackets in this protocol refer to the SPIRIT checklist item numbers. The order of the items has been modified to group similar items (see http://www.equator-network.org/reporting-guidelines/spirit-2013-statement-defining-standard-protocol-items-for-clinical-trials/).

**Table:** 

Title {1}	**Bathing Adaptations in the Homes of Older Adults (BATH-OUT-2): study protocol for a randomised controlled trial, economic evaluation and process evaluation**
Trial registration {2a and 2b}	ISRCTN48563324Prospectively registered on 09/04/2021
Protocol version {3}	Version 9.0 13.02.2023
Funding {4}	National Institute for Health Research – School for Social Care Research
Author details {5a}	Phillip Whitehead – Population Health Sciences Institute, Newcastle University Stuart Belshaw – c/o Centre for Rehabilitation and Ageing Research, University of Nottingham Samantha Brady – Department of Health Sciences, University of York Elizabeth Coleman—Department of Health Sciences, University of York Alexandra Dean – Department of Health Sciences, University of York Laura Doherty—Department of Health Sciences, University of York Caroline Fairhurst—Department of Health Sciences, University of York Sandra Francis-Farrell – Nottingham City Council Miriam Golding-Day – Centre for Rehabilitation and Ageing Research, University of Nottingham Joanne Gray – Department of Nursing, Midwifery and Health, Northumbria University Newcastle Maisie Martland – Department of Health Sciences, University of York Jennifer McAnuff – Department of Social Work, Education and Community Wellbeing, Northumbria University Newcastle Andrew McCarthy—Department of Nursing, Midwifery and Health, Northumbria University, Newcastle Peter McMeekin – Department of Nursing, Midwifery and Health, Northumbria University Newcastle Natasha Mitchell – Department of Health Sciences, University of York Melanie Narayanasamy – Centre for Rehabilitation and Ageing Research, University of Nottingham Craig Newman – Population Health Sciences Institute, Newcastle University Adwoa Parker – Department of Health Sciences, University of York Tim Rapley – Department of Social Work, Education and Community Wellbeing, Northumbria University Newcastle Sara Rodgers – Department of Health Sciences, University of York Leigh Rooney – Population Health Sciences Institute, Newcastle University Rachel Russell – Foundations Laura Sheard—Department of Health Sciences, University of York David Torgerson—Department of Health Sciences, University of York
Name and contact information for the trial sponsor {5b}	Sponsor: Newcastle University Sponsor Contact: Ms Kay Howes, Head of Faculty Research, Faculty of Medical Sciences, Newcastle University Newcastle upon Tyne, NE1 7RU Email: sponsorship@newcastle.ac.uk.
Role of sponsor {5c}	The study sponsor and funder have no role in the study design or collection, management, analysis or interpretation of the data. They will have no role in the writing of associated publications and the decision to submit papers for publication.

## Introduction

### Background and rationale {6a}

Appropriate housing plays an important role in maintaining health by supporting people to retain their independence, which in turn reduces the demand for health and social care services [1]. Housing adaptations, defined as ‘any permanent alteration carried out to a building with the aim of making it more suitable for a disabled person’ [2], were identified as a ‘top ten’ prevention intervention for older adults in an international review [3]. Housing adaptations may be beneficial in a number of ways. Adaptations, such as accessible showers, may enable people to manage their own personal care. This may alleviate the need for domiciliary care, where difficulties in recruitment mean the demand for carers may exceed the availability of such care [4]. Adaptations may also reduce falls which are the most common cause of injury-related deaths for people aged over 75 in the UK [5] with an estimated annual NHS cost over £2 billion [6]. However, there is a paucity of high-quality evidence on the health and care outcomes of housing adaptations. Additionally, there can be lengthy delays in provision, with some local authorities reporting that people may wait for 2 to 4 years [7], and further evidence is needed on the impact of delays on outcomes.

Disability in bathing for older adults is particularly important as onset can be rapidly followed by disability in other daily living tasks [8]; persistent difficulty in bathing is also associated with the risk of long-term nursing home admission [9]. A ‘bathing adaptation’ usually involves the removal of the bath and replacement with an accessible, ‘level-access’ shower and is the most common type of major housing adaptation for older adults. Such adaptations may restore the ability to bathe independently or enable a carer to support safe bathing. However, it is possible that older adults may start to experience difficulties with other daily living activities (such as dressing) while they are waiting for their adaptation. Furthermore, they may avoid leaving the house or attending social situations due to concerns about their personal hygiene [10]. This may lead to more rapid functional deterioration and further reduce the preventative effect. More academic research is vital to understand both the health and care outcomes of housing adaptations [11] and the impact of delays in provision.

A systematic review of the health effects of housing adaptations reported that there is good evidence for the effectiveness and cost-effectiveness of ‘minor’ adaptations such as grab rails and altered thresholds, which are especially effective in reducing injuries related to falls [12]. An RCT of ‘minor’ housing adaptations, conducted in New Zealand [13] found a 26% reduction in the rate of injuries caused by falls in the intervention group. However, the systematic review [12] also concluded that further research was needed to evaluate the health impacts of major adaptations in a UK context, such as accessible showers, and recommended the need for RCTs to be conducted.

We are not aware of any large RCTs that have focussed on ‘major’ housing adaptations, such as accessible showering facilities. We previously undertook a single-site feasibility RCT in one local authority area in the UK [14]. We created an expedited experimental group where we ‘speeded up’ the adaptation process for comparison with a waiting list control group who received standard waiting times. We demonstrated that recruitment, randomisation and intervention delivery within differing timescales were feasible. Participant outcomes improved across all measures following the adaptations, demonstrating the suitability of measures used [14] and an extended follow-up study suggested that the indicative benefits may last beyond the duration of our feasibility trial [15]. A nested qualitative interview study supported the use of our outcome measures and in particular the domains underpinning the Adult Social Care Outcomes Toolkit (ASCOT) [16] were found to be consistent with older adults’ lived experiences [10]. We also conducted a systematic review of interventions to promote independence in bathing (not confined to housing adaptations) [17] for older adults and identified only one comparative, non-randomised study highlighting the need for robust evidence of interventions to support bathing.

## Objectives {7}

BATH-OUT-2 aims to determine the effectiveness and cost-effectiveness of bathing adaptations compared to no adaptations and to explore the factors associated with routine and expedited implementation of bathing adaptations.

The objectives are as follows:

### Primary objective

To determine the effectiveness of bathing adaptations compared to no adaptations, using routine waiting times to form a control group, on the physical well-being of older adults as assessed by the Physical Component Summary (PCS) score of the Short Form-36 (SF-36) 4 weeks after the intervention group receive the adaptation.

### Secondary objectives

- To determine the effectiveness of bathing adaptations compared to no adaptations on the secondary outcomes of mental well-being, self-reported falls, health and social care services and resource use, health-related quality of life, social care-related quality of life, perceived risk of falling, independence in daily activities, independence in bathing and perceived difficulty in bathing.
- To carry out an extended follow-up of participants over a 12-month period in order to determine the effect of waiting times on outcomes and resource use.
- To conduct an economic evaluation to determine the cost-effectiveness of bathing adaptations compared to no bathing adaptations and expedited versus routine provision of bathing adaptations.
- To carry out a mixed-method process evaluation involving a range of stakeholders in order to explore and evaluate trial systems and process and the factors associated with the implementation of expedited and routine provision of bathing adaptations.

## Trial design {8}

BATH-OUT-2 is a multi-centre, two-arm, parallel-group, superiority RCT with pairwise allocation in a 1:1 ratio. The trial will involve embedded economic and process evaluations. The mixed methods process evaluation will draw on interviews with trial participants, trial decliners and social care and housing professionals as well as a survey of local authorities with social care responsibilities in England.

## Methods: participants, interventions and outcomes

### Study setting {9}

The study is set within local authority housing adaptation services in England. Participants will have been referred to the housing service with a recommendation for the provision of a level or easy-access shower adaptation. The intervention will be provided within the homes of older adults and is delivered through support from local authority housing adaptation services. Data will be collected from participants in their own homes using remote or face-to-face methods, as appropriate.

### Eligibility criteria {10}

We will recruit participants who fulfil the following criteria:

#### Inclusion criteria

1. People aged 60 or over.
2. People referred for a major adaptation for the provision of an accessible (level or easy access) showering facility. This may be by removal of an existing bath or shower cubicle.
3. People living in housing owned by the local authority or living in privately owned housing (owner occupied, privately rented, housing association owned) and who appear to be eligible for a Disabled Facilities Grant (DFG) and/or assistance from the local authority.

#### Exclusion criteria

1. People referred for an accessible showering facility *plus* one or more other major adaptations (e.g. ramps, hoists, lifts) as these adaptations are more complex and will involve extended timescales.
2. People referred for a rapid, fast-tracked or urgent priority bathing adaptation.
3. People who lack the mental capacity to provide informed consent and we are unable to identify a personal or nominated consultee.
4. People who lack the mental capacity to provide informed consent and who are unable to provide any study outcomes with support or where we are unable to identify an ‘alternative participant’ to provide data.

We will include people who do not speak English and will provide interpreters where required.

The process evaluation inclusion criteria are:

- People using the adaptation service
  1. Eligible to take part in the main trial (either consents or declines to do so).
  2. Ability to provide informed consent.
- Consultee
  1. Approached to act as a consultee for a person eligible for the main trial.
  2. Ability to provide informed consent.
- Alternative participant
  1. Approached to act as an alternative participant for a person eligible for the main trial.
  2. Ability to provide informed consent.
- Professional interview
  1. Social care or housing professional with responsibility for decision-making and/or delivery of bathing adaptation and/or associated processes (i.e. Disabled Facilities Grant administration).
- National survey
  1. Local authority in England with adult social care responsibility.

### Who will obtain informed consent? {26a}

People referred to the Local Authority for a bathing adaptation will initially be screened for eligibility by a member of staff at the local authority (the ‘site’). A member of staff at the site will screen every referral received for eligibility against the inclusion/exclusion criteria. The local authority staff member will contact potentially eligible people by telephone to provide a brief overview of the study and seek verbal agreement for the research team to make contact. If they are concerned that the potential participant does not understand the study, they will seek to gain consent to contact from a consultee (personal or nominated). A personal consultee can be a person who is engaged in the care and support of the potential participant or is interested in their welfare. If they are unable to identify a personal consultee, they will attempt to identify a nominated consultee in accordance with the Department of Health and Social Care’s guidance on identifying consultees for people who lack the capacity to consent [18]. If a consultee is needed but cannot be identified, they will not proceed with consent to contact. The primary outcome for this study is the Short Form 36 (SF-36) physical component summary score which cannot be completed on a participant’s behalf and therefore will not be collected from participants who lack the mental capacity to complete this. However, secondary outcomes such as the Barthel Index and the use of health and social care services and resources are very important for participants who lack the mental capacity to complete the SF-36 and can be collected by another person on the participant’s behalf. Bathing adaptations may enable the carer to manage and may potentially prevent the burden and expense of long-term care [9]. Normally, people who cannot complete the primary outcome are not recruited into a study. However, because these participants form an important part of the population who receive this service, they will be recruited into a stratified subgroup and an alternative participant will provide information on the participant’s behalf. Alternative participants can be the same person as the consultee and are solely required to answer questionnaires on the participant’s behalf.

Potential participants, consultees and alternative participants will be posted an information pack for the study, including a Participant Information Leaflet (PIL), a Summary Leaflet (a 1-page leaflet giving a brief overview of the study) and consent form (for information) with a letter inviting them to take part in the trial. The PIL will clearly state that the participant is free to withdraw from the study at any time for any reason without prejudice to future care, and with no obligation to give the reason for withdrawal.

Potential participants will be contacted by a researcher approximately 1 week after receiving the information pack. The researcher will confirm whether the potential participant has the capacity to consent, answer any questions that the potential participant has about study participation and complete a screening form to confirm eligibility. If the person would like some more time to make their decision, then the researcher will contact them at least 24 h after the initial consultation. All potential participants willing to proceed will be asked to provide audio-recorded verbal informed consent, unless circumstances allow for the consenting process to take place face to face, or if they express a preference for completing the paper version of the consent form.

Where the potential participant is unable to provide informed consent due to a lack of mental capacity, a consultee opinion shall be sought. A Consultee Information Leaflet and Consultee Form will be sent to an appropriate consultee in order to determine whether they believe that the potential participant would want to enter the study if they had the capacity to make the decision. Informed consent or consultee opinion will be collected from each participant before they take part in any study-related activity. The researcher will go through all items on the consent or consultee form and ask for the participant to agree, or consultee to give their opinion, verbally on the audio recording or sign in person. When consent is obtained, the researcher will go through the baseline questionnaire with the participant, with the help of another person if required. If the participant is unable to answer the questions, an alternative participant will be recruited and will complete a shortened questionnaire (see the ‘Outcomes {12}’ section). We will seek advice from the trial participant and the consultee about the most appropriate person to act as an alternative participant. The alternative participant will also undergo the consent process as above, completing an Alternative Participant Consent Form and receiving an Alternative Participant Information Sheet.

### Informed consent: process evaluation

The consent form for the main study will contain an optional statement about receiving information about taking part in an interview. People who decline to take part in the main study but agree to be contacted about an interview will be asked for verbal consent to have their details passed onto the process evaluation team. People using the service, or their consultees or alternative participants, who indicate they are willing to be approached to be interviewed and are chosen by the study team, will be approached up to 1 month after their main trial consent form is received. If the process evaluation team decide to approach a trial participant, a trial decliner or their consultee, or alternative participant for interview, they will be sent a separate, interview-specific PIL and informed consent form by the process evaluation team. The process evaluation team will then contact potential process evaluation participants within 10 days by telephone and go through the interview-specific PIL and answer any questions that they have. If the person agrees to take part in the interview, a separate appointment will be made for consent and interview. The timescale will be agreed between the researcher and the potential participant but will be at least 24 h after the initial study consultation. People using the service who are willing to take part will be offered the audio consent option, mirroring the consent process for the main trial, with a postal return of consent form as a second option, if preferred. Face-to-face recruitment will only commence when deemed safe to do so, in accordance with the main trial. The process evaluation team will contact potential participants by telephone to further discuss their participation. If the person decides to take part in the interview, they will then be offered a choice of method of the interview, either by telephone or online via an appropriate secure platform.

### Additional consent provisions for collection and use of participant data and biological specimens {26b}

This is not applicable. There are no biological specimens collected within the BATH-OUT-2 trial; therefore, additional consent for collection and use is not required.

### Interventions

#### Explanation for the choice of comparators {6b}

The comparators selected are a ‘bathing adaptation’ provided via the usual timescales of the local authority (control group) versus via an accelerated (expedited) process (intervention group). For the purpose of this study, a bathing adaptation is the provision of an accessible showering facility which usually involves replacing an existing bath with a flush floor, anti-slip, walk-in ‘level-access’ shower (or ‘wet room’). It may also include an easy-access shower or the alteration of an existing shower cubicle to make it more accessible. These comparators have been chosen to assess the effectiveness and cost-effectiveness of bathing adaptations compared to no adaptations (the primary objective) and to determine the effect of waiting times on the outcome measures (a secondary objective).

#### Intervention description {11a}

##### Control

Bathing adaptation within the usual timescale. The participant will undergo the usual process and timescales for receiving a bathing adaptation and be allocated to a project officer/surveyor to begin the adaptation process when they reach the top of the waiting list and/or by the usual processes and timescales within the local authority.

##### Expedited bathing adaptation

The participant will be allocated immediately to a project officer/surveyor to begin the adaptation process and/or will have their adaptation process expedited by active management of the process and rapid or fast-tracked contracting.

#### Criteria for discontinuing or modifying allocated interventions {11b}

The decision to discontinue the intervention or control may be made by the participant or in conjunction with the study team. Examples include the participant no longer requiring or wishing to receive a level-access shower or having a change of circumstance requiring they are moved to a rapid installation of their bathing adaptation. Withdrawals will be classed as one of the following:

- Full withdrawal
- Withdrawal from treatment
- Withdrawal from follow-up.

Participants lacking mental capacity will be withdrawn if they show signs of distress or any indication that they do not wish to take part in the study, and may be withdrawn by their consultee. If a participant loses capacity during the study, the study team would seek a consultee’s opinion as to whether the person should continue in the study, before collecting any further data. If a consultee’s opinion is not obtained, then the participant will be withdrawn from the study. If a consultee opinion is obtained, then the participant will continue according to the procedures for where we have obtained the consultee’s opinion.

#### Strategies to improve adherence to interventions {11c}

The intervention is the installation of a bathing adaptation so strategies have not been included to improve adherence as they are not applicable in this setting.

#### Relevant concomitant care permitted or prohibited during the trial {11d}

During the trial, concomitant medications and treatments will continue as per usual care.

#### Provisions for post-trial care {30}

At the end of the trial, participants will continue with their usual care from the local authority adult social care services and healthcare providers.

### Outcomes {12}

The use of pairwise randomisation means that the timing of the follow-up for each pair can be based on the completion of the bathing adaptations. For each randomised pair, participant follow-up data will be collected at three points: (1) 4 weeks after the intervention participant receives their bathing adaptation, (2) 4 weeks after the control participant receives their bathing adaptation or at 9 months (whichever is sooner) and (3) 12 weeks after the control participant receives their bathing adaptation, or at 12 months (whichever is sooner). For some participants recruited early in the study, data were collected 2 weeks before the planned installation date of the bathing adaptation in the control group participant, where possible; however, a protocol amendment was approved to remove this follow-up as the majority of participants were not completing this due to the dates of their showers being installed or difficulty getting the dates in time to schedule the follow-up. We will follow the majority of participants for 12 months; however, for the participants recruited at the end of the trial, we will follow them for 9 months (i.e., follow-up will continue for 9 months from the randomisation of the final participants).

#### Primary outcome

The primary outcome is in physical health status, measured using the Physical Component Summary (PCS) score of the SF-36 [19, 20]. The SF-36 is a generic measure of perceived health status and quality of life, which can be used across a range of medical conditions and disabilities. Its selection is based on the inclusion of the physical functioning subscale and is informed by the findings from the BATH-OUT-1 feasibility study [10, 14] and consultation with our Public and Person Involvement (PPI) group. The primary analysis will compare the PCS score of the SF-36 between the two groups, excluding the group lacking the mental capacity to complete it. The primary endpoint is the first follow-up assessment, which is 4 weeks post-adaptation in the intervention group, to evaluate the effect of adaptation versus no adaptation. Data from later time points will be used to address the secondary objectives.

#### Secondary outcomes

All secondary outcomes are collected at baseline and each of the follow-up time points:

1. Perceived mental health status measured via the Mental Component Summary score (MCS) of the SF-36 [19, 20].
2. Number of falls (self-reported).
3. Health and social care service use and associated costs (will be captured using a purposely designed and tested resource use questionnaire).
4. Health-related quality of life using the EuroQol EQ-5D-5L [21].
5. Social care-related quality of life using the Adult Social Care Outcomes Toolkit (ASCOT) [16].
6. Perceived risk of falling (Short Falls Efficacy Scale) [22].
7. Independence in bathing (bathing question of the Barthel Index) [23].
8. Ability to manage personal activities of daily living (Barthel Index) [23]. This analysis will include those who could not complete the SF-36 at baseline.
9. Perceived difficulty in bathing (0–100 scale).
10. Incremental cost per quality-adjusted life year (QALY) gained.

#### Alternative participants

If a consultee opinion is obtained for participation in the study, the extent to which the participant will answer the questions will be determined on a case-by-case basis and will be facilitated wherever possible. Participants may have support from another person (who may or may not be the consultee) as appropriate and any such support will be documented. In some cases, it will be clear that the participant cannot provide answers to questions. Participants who lack the mental capacity to complete participant self-reported outcome measures will complete a shortened assessment battery with an alternative participant. This assessment battery will include only factual information regarding the participant and the alternative participant’s opinions. These participants will not complete the primary outcome measure and we have accounted for this in our sample size calculation. We will collect the following secondary outcome measures at the follow-up time points previously stated where feasible:

1. Health-related quality of life using the EuroQol EQ-5D-5L Proxy Version 2 [24].
2. Health and social care service use and associated costs (this will be captured using a purposely designed resource use questionnaire).
3. Independence in bathing (bathing question of the Barthel Index) [23].
4. Ability to manage personal activities of daily living (Barthel Index) [23].
5. Number of falls.

### Participant timeline {13}

Participants will be enrolled in the study, and the baseline assessment completed, as soon as reasonably practicable after they have been referred to the housing adaptations service. The timing of outcome assessments is detailed in the previous section.

### Sample size {14}

Studies examining the utility of the SF-36 across different clinical conditions have estimated the Minimum Clinically Important Difference (MCID) to be between 3 and 7 points [25–27]. This trial is powered to detect a conservative MCID of 4 points for our target population of older adults with a range of clinical conditions. The standard deviation of the SF-36 PCS in BATH-OUT-1 at baseline was 8 [14]. To detect a standardised difference of 0.5 with 80% power and 5% two-sided alpha and allowing for up to 45% non-collection of primary outcome data, and the inclusion of up to 15% of participants who lack the capacity to provide the primary outcome (based on data presented in the December 2022 Trial Steering Committee meeting), we will aim to recruit a total of 272 participants.

### Recruitment {15}

Recruitment will initially be undertaken by local authorities. Potential participants will be approached by members of staff at the site. If the person consents to contact by a researcher, they will receive an information pack and a telephone call to provide more information about the study and will be recruited at this point if they wish to consent.

## Assignment of interventions: allocation

### Sequence generation {16a}

Eligible, consenting participants will be randomly allocated 1:1 to either the intervention (expedited bathing adaptation) or the usual care control group using the York Trials Unit secure web-based randomisation system. We will use ‘pairwise’ randomisation, at the level of the participant. Randomisation will be stratified by the capacity to complete the primary outcome. For those strata where the participant can complete the primary outcome, the randomisation is also stratified by site and property tenure (owner-occupied, local authority owned, privately rented including private and social landlords as one group), and for those who cannot, it will be stratified by site only. Randomisation will be stratified in this way as the primary analysis will exclude the group that was identified as being unable to complete the primary outcome at randomisation, and also as the wait for bathing adaptation can vary across sites and property tenures. After two participants within the same strata are recruited, they will be randomised together such that one will be allocated to the intervention group and the other to the control group, in a random order. We will do this so that we can follow both members of the pair up at the same time post-receipt of their adaptation to reduce the confounding effect of time to assessment. This is not the same as ‘matched’ randomisation.

Depending on the speed of recruitment, there is a risk that eligible participants may be left waiting to be randomised until another participant is available to pair with them. We shall limit the time participants wait to be randomised by allowing some flexibility for them to be paired, for example, with someone from a different property tenure within the same site and capacity. If a participant has not been randomised 3 weeks after consenting, they will be paired and randomised with any other participant who has consented at the same site. If, after 6 weeks, a participant has not been randomised, they will be paired and randomised with any other participant who has the capacity and has consented at any site. For those participants who are deemed not able to complete the primary outcome, they may be randomised using simple randomisation—without a pair, should the time waiting for a pair be too long.

### Concealment mechanism {16b}

Randomisation for the main trial will be completed by a central secure randomisation service hosted by the York Trials Unit, University of York. Randomisation will be completed via the Internet, with information recorded to check eligibility prior to randomisation. The randomisation system is designed and maintained by an independent data systems manager at the York Trials Unit, who is not involved in the recruitment of participants.

### Implementation {16c}

The allocation sequence for the main trial will be generated by the trial statistician who is independent of the recruiting sites and study researchers. This sequence will be implemented using the secure randomisation service that can be accessed by staff at the York Trials Unit and will assign participants to either expedited bathing adaptation or usual care.

## Assignment of interventions: blinding

### Who will be blinded {17a}

Researchers involved in obtaining informed consent and collecting data will not be informed of participant group allocation and will be blinded, as far as possible. However, due to the nature of data collection, it is not possible to ensure blinding. It will not be possible to conceal the allocation from the participants or from the adaptations or social care staff. The research team at YTU will also be aware of the allocation of participants for the purpose of the management of the trial.

### Procedure for unblinding if needed {17b}

Researchers who are unblinded to a participant’s allocation will be asked to record the source and details of the unblinding. Given the low-risk nature of this trial, and that the participant, coordinating centre and local authority will know the participant’s allocation, there will be no other circumstances where unblinding will be possible.

## Data collection and management

### Plans for assessment and collection of outcomes {18a}

**Fig. 1:**
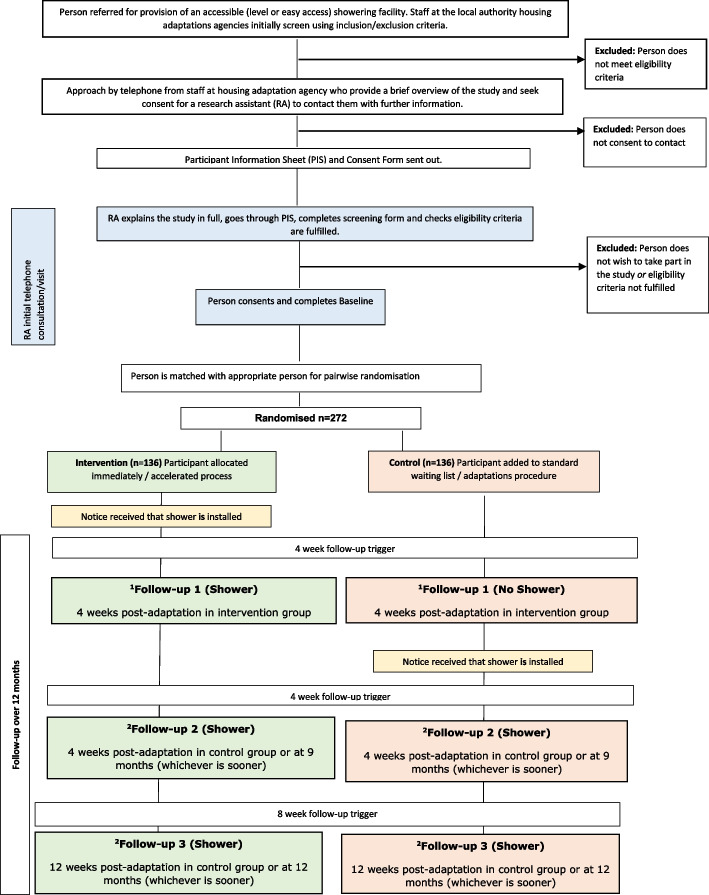
Participant flow through the study

**Fig. 2:**
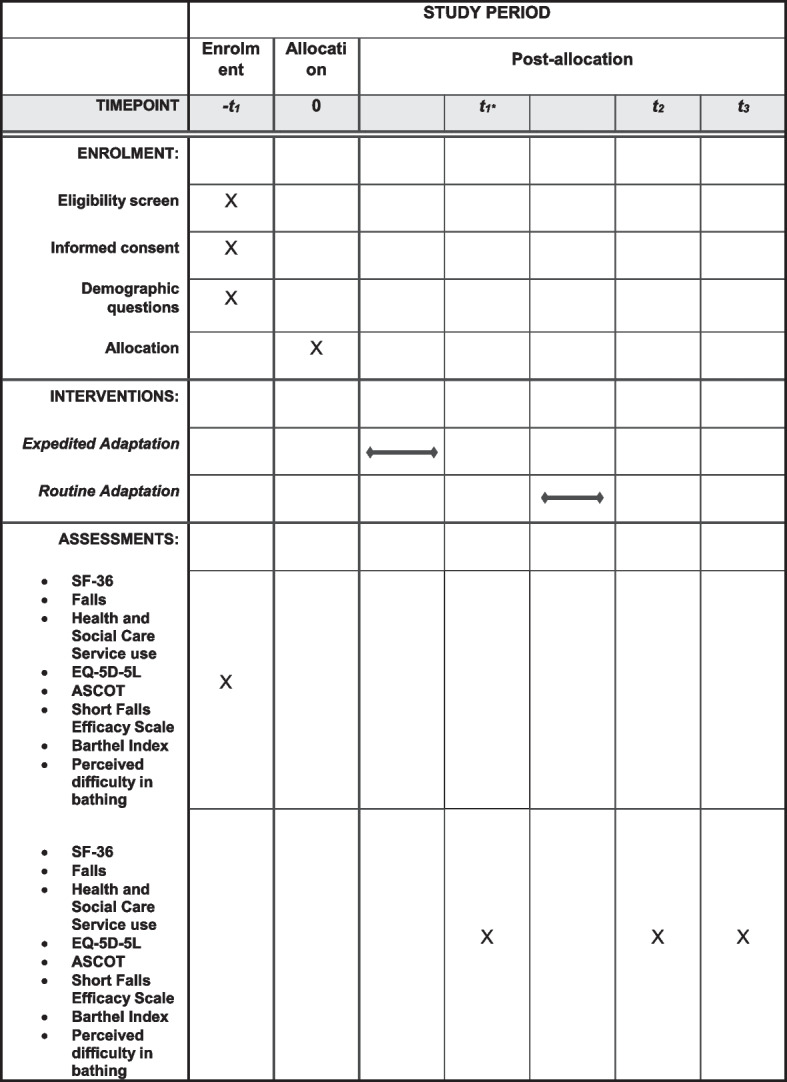
Schedule of enrolment, interventions and assessment. *Some participants enrolled early in the trial had an additional follow-up between t1 and t2 before the intervention was provided in the routine adaptation group. This follow-up has now been removed from the current version of the protocol

Data will be collected at the following time points for both members of each pair (please see Figs. 1 and 2): (1) 4 weeks after the intervention participant receives their bathing adaptation, (2) 4 weeks after the control participant receives their bathing adaptation, or at 9 months (whichever is sooner) and (3) 12 weeks after the control participant receives their bathing adaptation or at 12 months (whichever is sooner).

At baseline, participants who are able to answer questions themselves will complete a questionnaire comprised of participant demographics, property tenure, COVID-19 status and several outcome measures: the SF-36 [19, 20], number of falls, a health and social care service and resource use questionnaire, the EQ-5D-5L [21], the ASCOT questionnaire [16], the short Falls Efficacy Scale (FES) [22], the Barthel Index [23] and the bathing question of the Barthel Index [23]. If the participant lacks mental capacity, the alternative participant will complete a shortened questionnaire comprising participant demographics, property tenure, COVID-19 status and several outcome measures including number of falls, a health and social care questionnaire, the EQ-5D-5L (proxy version 2) [24], the Barthel Index [23], the bathing question of the Barthel Index [23] and perceived difficulty in bathing.

Similarly, at the three subsequent data collection time points, participants who are able to answer questions themselves will complete a questionnaire comprised of the SF-36 [19, 20], number of falls, a health and social care service and resource use questionnaire, the EQ-5D-5L [21], the ASCOT questionnaire [16], the short FES [22], the Barthel Index [23] and the bathing question of the Barthel Index [23]. If the participant lacks mental capacity, the alternative participant will again complete shortened questionnaire comprising a number of falls, a health and social care questionnaire, the EQ-5D-5L (proxy version) [24], the Barthel Index [23], the bathing question of the Barthel Index [23] and perceived difficulty in bathing.

Participants will be sent a ‘diary’ to complete following the first follow-up visit and after each subsequent follow-up visit to enable them to record contacts with health, social care and other services. This diary will be optional, for them to complete at home. The purpose is to aid their recall where there is a longer time period between each follow-up. The diary will not be returned to the research team.

### Plans to promote participant retention and complete follow-up {18b}

Participants will be sent a newsletter 6 months post-randomisation to maintain contact and promote engagement. The newsletter will be produced in collaboration with the PPI group and will thank participants for their time and involvement and have an FAQ section for possible queries that may arise. Where possible, we will aim to retain participants in the study for follow-up if they withdraw from the intervention.

### Data management {19}

All participant source data collected will be recorded on case report forms (CRF). This will be centrally monitored by the University of York’s Trials Unit. To ensure high-quality data, data collected within the case report forms will be processed at the York Trials Unit, using a licensed, automated, electronic system (Teleform) which allows data to be entered, checked and validated. Further details pertaining to the processing of the data will be documented in a study-specific data management plan.

Study documentation (both paper and electronic) will be retained in accordance with Good Research Practice and UK Law for 5 years after study completion in the Trial Master and Investigator Site Files, after which time information will be securely destroyed.

### Confidentiality {27}

Participants will be allocated a unique pseudo-anonymised ID number that they will keep throughout the trial.

Copies of paper documentation containing personal data will be stored at York Trials Unit, Newcastle University and the University of Nottingham. All data will be stored in a locked cupboard in a locked room with access to the cupboard restricted to the study team only. Identifiable and non-identifiable information will be stored separately. The key to the cupboard will be held by a data manager at York Trials Unit or designated study personnel at other study locations. Data stored electronically will only be accessible by password and only by individuals directly involved in the study.

### Plans for collection, laboratory evaluation and storage of biological specimens for genetic or molecular analysis in this trial/future use {33}

Not applicable. There are no biological specimens being collected in this trial.

## Statistical methods

### Statistical methods for primary and secondary outcomes {20a}

#### Statistical analysis

A detailed statistical analysis plan will be agreed upon with the joint Trial Steering Committee (TSC) and Data Monitoring Committee (DMC) prior to the completion of data collection. All analyses will be conducted using the principles of intention to treat (ITT), including all available randomised participants in the groups to which they were allocated, where data are available. Analyses will be conducted using two-sided statistical tests at the 5% significance level. The flow of participants through each stage of the trial will be presented in a CONSORT diagram. Baseline and outcome data will be summarised descriptively by treatment group and overall.

The primary analysis will compare the PCS score of the SF-36 [19, 20] between the two groups, excluding the group lacking the mental capacity to provide this, using a covariance pattern mixed linear regression model incorporating all post-randomisation time points, where effects of interest and baseline covariates are specified as fixed effects, and the correlation of observations within patients over time (random effect) is modelled by a covariance structure. The model will adjust for the treatment group, time, treatment group-by-time interaction, baseline PCS score and property tenure, with participant and site as random effects. Estimates of the difference between treatment groups in PCS scores will be extracted for all time points with a 95% confidence interval and *p*-value. The primary endpoint will be the treatment effect estimate at the first assessment time point (4 weeks post-fitting of the intervention participant adaptions) to evaluate the effect of adaptation versus no adaptation. We will also consider a linear regression analysis that compares data from the two groups taken 4 weeks after their shower adaptations have been installed (first follow-up for intervention group participants, second follow-up for those control group participants who have received their adaptation). This will allow us to investigate whether increased wait times impact the short-term effect of the intervention.

Other continuous secondary outcomes (e.g. SF-36 MCS, ASCOT, Barthel Index and FES-I) will be analysed using the same methods as the primary analysis; however, these analyses will also include those who could not complete the SF-36 at baseline. The number of falls experienced over the follow-up period will be compared between the groups using negative binomial regression adjusting for site and property tenure, and accounting for length of follow-up.

#### Health economic analysis

Both cost-effectiveness and cost-utility analyses will be conducted from personal health and social care services and a societal perspective including carers and paid care workers. The cost-utility analysis will be based on EQ5D-5L quality-adjusted life years (QALYs) and both will estimate costs. Secondary cost-effectiveness will be conducted using the Adult Social Care Outcomes Toolkit (ASCOT). Our analysis will take both personal health and social services and a societal perspective as we expect costs and savings will also accrue to family members. Incremental cost-effectiveness ratios (ICER) and cost-effectiveness acceptability curves (CEACs) will be produced using each outcome measure for the expedited adaptations versus usual waiting time control, at each time point and uncertainty will be explored.

Furthermore, aiming to strengthen the alignment between data analysis and decision-making processes, we will estimate the return on investment (ROI) for expedited adaptations versus usual waiting time control and build a ROI tool for use by decision-makers involved in commissioning and provision in home adaptation services. ROI is a form of economic evaluation that values the financial return, or benefits, of an intervention against the total costs of its delivery [28]. This ROI tool will extend the work conducted regarding the ROI for housing adaptations in relation to falls on stairs (reported in Powell et al. [12] and will estimate the ROI for improved waiting times and reduced delay. The benefit minus the cost expressed as a proportion of the cost will be estimated and the results will be used to parameterise a ROI toolkit using Microsoft® Excel [29]. This spreadsheet-based toolkit will estimate the costs and consequences of improving waiting times according to local characteristics and assumptions, such as prevalence and uptake of adaptations.

### Interim analyses {21b}

There are no planned interim analyses and no planned stopping rules for this trial.

### Methods for additional analyses (e.g. subgroup analyses) {20b}

A sensitivity analysis will be conducted, excluding any participants who indicated that they had COVID-19 within the course of the trial follow-up period to explore the potential effects that COVID-19 may have had on the outcomes.

#### Process evaluation

Process evaluation qualitative data analysis will be ongoing and iterative throughout the trial. The analysis will be theoretically informed by Normalisation Process Theory (NPT) [30–32] and will be conducted according to the standard procedures of rigorous qualitative analysis [33] including open and focused coding, constant comparison, memoing [34], deviant case analysis [35] and mapping [36]. Independent coding and cross-checking will be completed and a proportion of data will be analysed collectively in ‘data clinics’ where the process evaluation research team shares and exchanges interpretations of key issues emerging from data.

Process evaluation survey data analysis will use descriptive statistics with results presented in text and tables and an accompanying narrative summary of findings. We will draw on a range of implementation frameworks and theories, including Theoretical Domains Framework [37] and NPT in order to identify determinants, to match them to strategies and for the implementation of trials in local authorities and to support the design and delivery of those strategies to implementation of bathing adaptations.

Across all aspects of the study, initial findings will be shared with the PPI group to determine their perspective and combine that with the findings from the research team.

### Methods in analysis to handle protocol non-adherence and any statistical methods to handle missing data {20c}

We anticipate that the amount of missing data will be relatively small as a researcher will be collecting the data from most participants. The impact of missing primary outcome data will be minimised to some extent by using the repeated measures model, which allows the inclusion of intermittent responders. PCS scores for complete and intermittent responders will be compared descriptively. The impact of missing data will additionally be assessed using multiple imputation by chained equations.

At the primary time point, there may be some non-compliance, e.g. participants in the intervention group who have not received their adaptation and some in the control group who have. We will summarise the time taken to receive the adaptations in each group and whether this was completed as per the protocol. The primary analysis will follow ITT, but we will conduct a sensitivity analysis using complier average causal effect (CACE) analysis to estimate the effect of the receipt of, rather than the offer of, the allocated treatment. A two-stage instrumental variable (IV) approach will be used using the randomised group as the IV.

### Plans to give access to the full protocol, participant-level data and statistical code {31c}

The datasets generated and/or analysed during the current study will be available upon reasonable request from the Chief Investigator following the completion of the trial and publication of trial results. Requests will be considered by the Trial Management Group on a case-by-case basis. Data will be made available for secondary analyses, and only anonymised data will be provided.

## Oversight and monitoring

### Composition of the coordinating centre and trial steering committee {5d}

York Trials Unit is acting as the coordinating centre for this study. This comprises a Trial Manager, Trial Coordinator, Trial Support Officer, Statisticians and Data Managers who will work alongside the Chief investigator and the Sponsor. The coordinating team will be responsible for ensuring all relevant approvals are in place, training and supporting sites to undertake the study and putting measures in place to obtain accurate data. The data management team will process and check data against the validation criteria agreed upon.

The Trial Management Group (TMG) comprising the coordinating team, public representative, statisticians, health economists, qualitative researchers, housing experts and other stakeholders involved in the trial will meet monthly to review trial progress.

The Trial Steering Committee (TSC) comprises social care researchers, housing adaptations experts, an independent statistician, an independent health economist and public members. The TSC will meet every 6 months to review trial progress.

The Public and Person Involvement (PPI) Group will meet quarterly to comment and collaborate on trial progress, proposed amendments and study documents such as the participant newsletter.

### Composition of the data monitoring committee, its role and reporting structure {21a}

Due to the low-risk nature of this trial, we have combined the role of the TSC with that of the DMC.

### Adverse event reporting and harms {22}

We do not anticipate any adverse events as part of this research. The intervention is an earlier provision of an intervention that would be provided later under routine care. Whilst we are collecting information on falls and hospital admissions, we are not expecting these to be related to the intervention and we will not assess them for relatedness to the study. Thus, no adverse events (or serious adverse events) will be reported for this study.

### Frequency and plans for auditing trial conduct {23}

No on-site monitoring will be conducted in this trial. Ongoing in-house quality checks and continuous monitoring by the coordinating centre will be applied. Annual compliance checks of sites will also be carried out by the York Trials Unit.

### Plans for communicating important protocol amendments to relevant parties (e.g. trial participants, ethical committees) {25}

Any amendments will be applied through REC as per standard practice. Recruiting sites and other relevant parties will be informed via email and all associated documentation passed on. In the event that participants need to be notified, a letter would be prepared and sent alongside the amendment to the REC for approval.

## Dissemination plans {31a}

We will aim to engage academic, stakeholder and lay audiences through publications and knowledge exchange activities during the course of this study.

We anticipate producing four high-quality, high-impact peer-reviewed papers (the study protocol; the main RCT findings, and cost-effectiveness findings; an additional complementary health economics paper exploring EQ5D and ASCOT QALYs; and the findings from the process evaluation). We have produced a commentary paper on the issues we have encountered in setting up the trial in a non-traditional setting (at the interface between adult social care and housing services within local authorities) for publication alongside the protocol [38]. Additionally, we will co-produce a brief Plain English Summary of the study results with our PPI group for study participants.

We will carry out a range of knowledge exchange activities throughout the duration of the project using both traditional and innovative methods. A fundamental and important knowledge exchange activity will be the production of a key finding infographic for older and disabled people, their families and carers, produced collaboratively with Foundations [39] and our PPI group. We will also carry out a range of less formal presentations to a variety of local authorities and other stakeholders, building on our success from BATH-OUT-1. During the set-up phase, we will complete presentations about the research at each of our local authority sites. We will also hold a national ‘results reveal’ dissemination event at the end of the study, in partnership with Foundations. This will also target commissioners of housing adaptations and will launch our return on investment tool. Other outputs will be disseminated through our extensive networks with dissemination activities targeting policymakers in national and local government; commissioners operating at the interface of housing, health, public health and social care; and practitioners involved in the delivery of home adaptations. We will also aim to present the study at a range of conferences.

## Discussion

To our knowledge, this is the first large, multicentre RCT to be undertaken in this local authority setting at the interface between social care and housing services. The relative novelty of RCTs in local authority settings has meant that there has been minimal prior infrastructure or processes established to support trial conduct, which has presented additional challenges [38]. For instance, unlike trials undertaken in the NHS which have standardised approved research contract templates, no prior research contract templates existed in the UK that could be used for recruiting local authorities for this trial. Therefore, the set-up process for sites has sometimes involved lengthy negotiations, and each local authority has required a slightly different contract.

The COVID-19 pandemic has presented additional challenges. We originally planned to recruit and follow up participants face to face in their homes; however, due to the pandemic and our study population being older and more vulnerable to COVID-19, we had to change to remote recruitment and data collection methods—by telephone or video depending on each participant’s preference. Although this has introduced new challenges, it has also meant we have been able to be more flexible with regard to the number and location of new study sites.

Although sites are undertaking major adaptations, their operations have been affected by the COVID-19 pandemic. The global impact of the pandemic on supply chains means that there have been greater than anticipated delays in obtaining adaptation supplies, as well as reduced staff to support the adaptation process. This has led to longer waits—both in the intervention and control groups.

Notwithstanding the above challenges, local authorities need evidence of the effectiveness of new and existing social care and housing interventions that seek to promote independence for older adults and delay the need for other services. Therefore, trials of major housing adaptations in the UK are required [12]. This RCT builds on our earlier feasibility work to undertake a large RCT. The findings of this study will be relevant to researchers, clinicians, commissioners, service users and carers and have the potential to highlight, and then reduce, health inequalities associated with waiting times for bathing adaptations.

## Trial status

The current version of the protocol is Version 9.0 13.02.2023. The start date for recruitment was 14 September 2021 and recruitment will end in August 2023. Follow-up will continue for 9 months from the randomisation of the final participants, until May 2024. We expect results to be available in late 2024.

## Data Availability

The datasets generated and/or analysed during the current study will be available upon reasonable request from the Chief Investigator following the completion of the trial and publication of trial results. Requests will be considered by the Trial Management Group on a case-by-case basis. Data will be made available for secondary analyses, and only anonymised data will be provided.

## Abbreviations

ADASS: Association of Directors of Adult Social Care Services
ADL: Activities of daily living
AE: Adverse event
ASCOT: Adult Social Care Outcomes Toolkit
BATH-OUT-1: Bathing Adaptations in the Homes of Older Adults 1: a feasibility study and nested qualitative interview study
BATH-OUT-2: Bathing Adaptations in the Homes of Older Adults: a randomised controlled trial, economic analysis and process evaluation
CI: Chief investigator
CEA: Cost-effectiveness analysis
CF: Consent form
CONSORT: Consolidated Standards of Reporting Trials
CRF: Case report form
CUA: Cost-utility analysis
DFG: Disabled Facilities Grant
DMC: Data Monitoring Committee
EQ-5D-5L: EuroQol Quality of Life, 5 domains, 5 levels
GCP: Good Clinical Practice
ICF: Informed consent form
ITT: Intention-to-treat
IV: Instrumental variable
MCID: Minimum clinically important difference
MCS: Mental Component Summary, of the Short Form 36
NIHR: National Institute for Health and Care Research
NHS: National Health Service
NPT: Normalisation process theory
OT: Occupational therapy/therapist
PCS: Physical Component Summary, of the Short Form 36
PI: Principal investigator at a local centre
PIL: Participant Information Leaflet
PPI: Public and Person Involvement
REC: Research Ethics Committee
RCT: Randomised controlled trial
R&D: Research and Development department
SAE: Serious adverse event
SSCR: School for Social Care Research
SF-36: Short Form 36
TMG: Trial Management Group
TSC: Trial Steering Committee
YTU: York Trials Unit
